# Metabolic reprogramming as a key regulator in *Helicobacter* *pylori*-infected gastric cancer

**DOI:** 10.1007/s10120-025-01675-x

**Published:** 2025-10-31

**Authors:** Ruofan Cao, Feifei Zhou, Cuiyu Zhu, Hongwei Xu

**Affiliations:** 1 Department of Gastroenterology, Shandong Provincial Hospital Affiliated to Shandong First Medical University, Jinan, Shandong 250021 People’s Republic of China; 2 Medical Science and Technology Innovation Center, Shandong First Medical University & Shandong Academy of Medical Sciences, Jinan, Shandong 250021 People’s Republic of China

**Keywords:** *Helicobacterpylori*, Gastric cancer, Metabolic reprogramming, Therapy

## Abstract

*Helicobacter* *pylori* (*H. pylori*) infection is a recognized risk factor for gastric cancer (GC), which is the leading cause of cancer-related deaths worldwide. As a Class I carcinogen, *H. pylori* plays a central role in the occurrence and development of GC. Recent studies have highlighted the critical role of metabolic reprogramming inthe gastric cancer, and *H. pylori* infection has been shown to significantly alter metabolic pathways in gastric cancer. This review explores the mechanisms by which *H. pylori* infection drives metabolic changes in GC, particularly in glycolysis, lipid metabolism, and amino acid metabolism. By altering these metabolisms, *H. pylori* enhances the survival, proliferation, and metastasis of tumor cells, and also promotes immune evasion. Therefore, understanding the ways in which *H. pylori*-induced metabolic reprogramming of GC cells is essential for identifying new therapeutic targets. By summarizing the latest research progress of these metabolic pathways, new strategies and directions can be provided for gastric cancer treatment.

**Graphical abstract:**

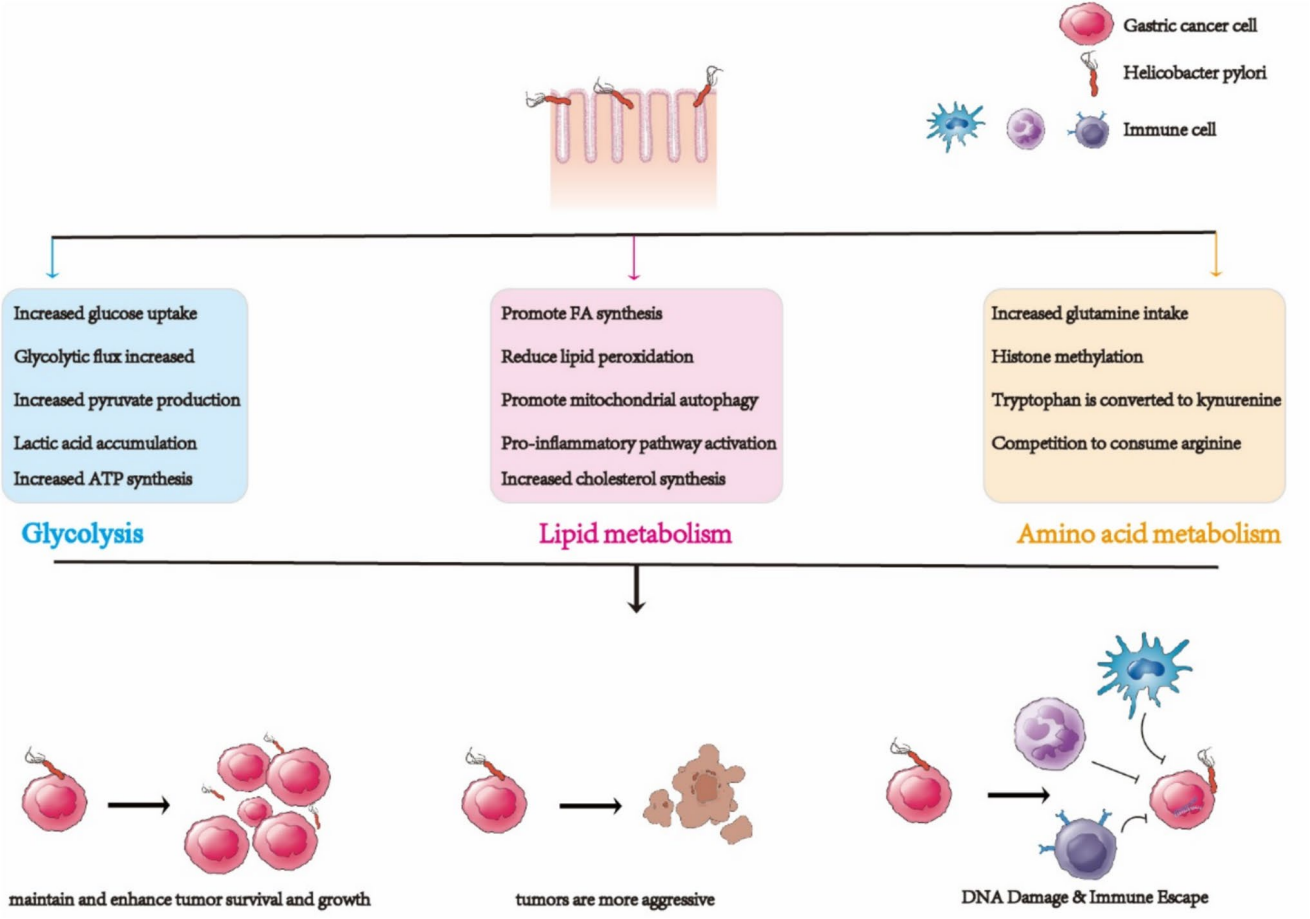

*H. pylori* regulates glycolysis, amino acid, and lipid metabolism to facilitate the occurrence and development of gastric cancer.

## Introduction

Infection with *H. pylori*, a ubiquitous pathogen that affects nearly half of the global population, is one of the most widespread bacterial infections worldwide [1]. For decades, it has been recognized as a major driver of various gastric ailments, from chronic gastritis to peptic ulcers, and most alarmingly, GC. GC is the fourth leading cause of cancer-related deaths worldwide, with nearly 660,000 people dying from the disease in 2022 alone. GC is an extraordinarily complex and multifactorial disease, influenced by genetic, environmental, and microbial factors [2–4]. Among these, *H. pylori* infection emerges as a prominent risk factor, playing a critical role in the development of gastric cancer. As a Class I carcinogen, *H. pylori* infection serves as a key initiator of gastric mucosal carcinogenesis, advancing through several stages, including chronic non-atrophic gastritis, atrophic gastritis, intestinal metaplasia, dysplasia, and ultimately, GC [5–7].

The relationship between metabolism and cancer was first noted by Warburg in the early twentieth century, when he discovered that tumor cells primarily rely on aerobic glycolysis rather than oxidative phosphorylation for their energy needs [8]. In recent years, this insight has expanded into a broader understanding of metabolic reprogramming as a central mechanism in cancer biology. Increasing evidence now highlights the pivotal role of metabolic reprogramming in the development of *H. pylori*-associated gastric diseases, including GC [9]. By altering cellular metabolism, cancer cells enhance their survival, promote metastasis, develop resistance to therapies, and evade immune detection. Although the precise mechanisms underlying these alterations remain under investigation, it is evident that *H. pylori* infection does more than merely support cell survival. It actively inhibits apoptosis, allowing the tumor to adapt and thrive in the hostile gastric microenvironment [10].

Hence, in this review, we explore the relationship between *H. pylori* infection and GC, focusing on how *H. pylori* regulates metabolic reprogramming in GC cells and recent advances in targeting these metabolic pathways for the treatment of GC.

### Association between *H. pylori* and GC

*H. pylori*, a Gram-negative bacterium that colonizes the human stomach, is widely recognized as a principal risk factor for GC, joining alcohol consumption and smoking as key contributors to this malignancy [11]. The World Health Organization (WHO) has even classified *H. pylori* as a Group 1 carcinogen, underscoring its dangerous potential in the development of GC [12]. Infection with this bacterium typically sets off a cascade of events, starting with chronic non-atrophic gastritis that, over time, can progress into more severe stages, such as intestinal metaplasia, dysplasia, and ultimately, GC [13]. This path of transformation highlights the importance of *H. pylori* eradication, which is not only a preventive measure against gastric carcinogenesis, but also a crucial strategy for halting the progression of GC, placing it at the forefront of preventive and therapeutic approaches.

Epidemiological data reveal that *H. pylori* infection is notably more prevalent in low- and middle-income countries, with significant concentrations in regions such as Africa, the Eastern Mediterranean, Russia, and parts of Central and South America. Strikingly, these high infection rates often overlap with areas where the incidence of GC is equally alarming, suggesting a strong correlation between *H. pylori* infection and the incidence of GC [14]. However, it is essential to highlight that the majority of individuals harboring *H. pylori* will not develop GC. Studies indicate that only a small fraction—approximately 2–3%—of infected individuals will develop gastric adenocarcinoma, while a mere 0.1% might go on to develop mucosa-associated lymphoid tissue (MALT) lymphoma [15, 16]. This disparity emphasizes the multifaceted nature of the carcinogenic process, where host factors, bacterial strain differences, and environmental influences all play vital roles in determining the ultimate risk of cancer development [17].

At the molecular level, several virulence factors of *H. pylori* have been implicated in the pathogenesis of GC, most notably the cytotoxin-associated gene A (CagA) and the vacuolating cytotoxin A (VacA) [18]. The cagA gene is more commonly found in the more virulent strains of *H. pylori*. Once the CagA oncoprotein is injected into host cells via a type IV secretion system (T4SS), encoded by the cag pathogenicity island (cagPAI), it sets off a series of events that lead to cytoskeletal rearrangements, disrupt cell signaling pathways, and ultimately result in enhanced cell proliferation and reduced apoptosis [19]. Phosphorylated CagA interacts with multiple host proteins, continuously activating carcinogenic pathways that contribute to the malignant transformation of gastric cells [20].

Alongside CagA, the VacA toxin secreted by *H. pylori* plays a critical role in the development of GC. VacA induces vacuolization within gastric epithelial cells, triggering apoptosis [21]. Capurro et al. revealed that VacA targets the lysosomal calcium channel transient receptor potential mucolipin 1 (TRPML1), disrupting lysosomal function and interfering with autophagic pathways. This disruption not only helps *H. pylori* evade the effects of antibiotics but also fosters a chronic inflammatory environment within the stomach, exacerbating tissue damage. As this inflammation persists, it facilitates the accumulation of genetic mutations and epigenetic alterations in gastric epithelial cells, a key step in malignant transformation [22].

Infected gastric tissues also exhibit upregulation of a variety of pro-inflammatory cytokines, including IL-1β, IL-6, IL-8, TNF-α, and NF-κB, alongside T helper (Th) type 1 cytokines (e.g., IFN-γ) and Th17-type cytokines (e.g., IL-17A, IL-21) [21, 23]. These inflammatory mediators not only aggravate local tissue damage but also drive the proliferation of cells, mutagenesis, oncogene activation, and angiogenesis. The longer the *H. pylori* infection persists, especially in the presence of complications such as chronic gastritis and atrophic gastritis, the greater the risk of developing GC.

Beyond its inflammatory effects, recent studies have increasingly highlighted the role of metabolic reprogramming in *H. pylori*-induced gastric carcinogenesis [24–26]. As gastric tumorigenesis progresses, *H. pylori* profoundly reshapes the metabolic landscape of gastric cells, influencing key metabolic processes such as glucose metabolism, lipid metabolism, glutamine metabolism, oxidative phosphorylation, and mitochondrial respiration [27]. Tumor cells undergo metabolic reprogramming to adapt to the changing tumor microenvironment (TME), supporting their growth and proliferation [28]. Thus, understanding how *H. pylori* orchestrates metabolic shifts in glycolysis, lipid, and amino acid metabolism is not only pivotal for comprehending its role in GC development but also for pinpointing novel therapeutic targets that could reshape the treatment of this devastating condition.

### The key role of metabolic reprogramming in GC

Metabolic reprogramming stands as one of the defining hallmarks of cancer cells, a crucial adaptation that enables them to thrive within the TME and sustain their rapid, unchecked growth, proliferation, and survival [10]. This metabolic flexibility allows cancer cells to continuously meet the heightened demands for energy and biosynthesis, all while driving the initiation and progression of tumors [29]. Increasing evidence highlights that alterations in fundamental metabolic pathways, such as glycolysis, lipid metabolism, and amino acid metabolism, promote aggressive tumor expansion and resistance to therapeutic intervention [30, 31].

One characteristic feature of metabolic reprogramming in GC is the shift toward aerobic glycolysis, commonly referred to as the Warburg effect. This phenomenon is characterized by an enhanced uptake of glucose and its subsequent conversion to lactic acid, even when oxygen is readily available [10]. In contrast to normal cells, which predominantly rely on oxidative phosphorylation for adenosine triphosphate (ATP) production under aerobic conditions, cancer cells prioritize glycolysis, a process that enables them to rapidly generate energy. This metabolic reorientation not only ensures an almost immediate supply of ATP but also generates a wealth of intermediates that fuel crucial biosynthetic pathways, such as nucleotide, lipid, and amino acid synthesis. Moreover, by embracing this glycolytic phenotype, cancer cells reduce oxidative stress, a key factor in ensuring their proliferation and survival in the hostile gastric environment [32].

Another critical aspect of metabolic reprogramming in GC is abnormal lipid metabolism [33]. Tumor cells are adept at remodeling lipid pathways, harnessing the diverse roles of lipids in cellular processes to escape the therapeutic pressures they face [34]. Abnormal activation of lipid biosynthetic pathways, such as the upregulation of genes involved in fatty acid synthesis or oxidation, is directly associated with malignant behaviors, including metastasis, drug resistance, and recurrence [35, 36]. Furthermore, these aberrant lipid metabolic processes influence a range of oncogenic signaling pathways, reshape the TME, and help tumor cells navigate metabolic stress by maintaining energy homeostasis and redox balance [37, 38]. Collectively, these changes enable GC cells to bolster their adaptability and aggressiveness, reinforcing their capacity to endure under adverse conditions.

Amino acid metabolism also plays an indispensable role in the progression of GC. Amino acids, the building blocks of proteins, are crucial for a variety of cellular functions [39]. Glutamine is a particularly important amino acid that is a key substrate for energy production and biosynthesis [40]. The enhanced catabolism of glutamine has emerged as a hallmark of cancer cell metabolism, driving rapid tumor growth and supporting the high metabolic demands of cancer cells [41, 42]. Similarly, alterations in tryptophan metabolism are associated with cancer progression and have important implications for immune regulation and cellular homeostasis [43]. These metabolic shifts not only facilitate tumor proliferation but also enhance the resilience of cancer cells against environmental challenges and therapeutic pressures.

In conclusion, metabolic reprogramming such as glycolysis, lipid metabolism, and amino acid metabolism provides GC cells with the energy and nutrients they need to survive. Targeting these pathways holds promise for the development of novel therapeutic strategies aimed at disrupting the metabolic dependencies of tumor cells, thereby improving treatment outcomes for GC patients [10, 44, 45].

## Metabolic reprogramming regulated by *H. pylori* in GC

### *H. pylori* and glycolysis

One of the critical metabolic changes induced by *H. pylori* infection is the reprogramming of energy metabolism, particularly the enhancement of glycolysis, even in the presence of oxygen. Recent studies have shed light on the intricate molecular mechanisms that fuel this metabolic shift, providing deeper insight into how this bacterium manipulates host cellular processes to promote GC progression.

*H. pylori* infection is known to upregulate Class E basic helix–loop–helix protein 40 (BHLHE40) [46], which subsequently drives the expression of glutamate ionotropic receptor N-methyl-D-aspartate type subunit 2D (GRIN2D) in GC cells [25]. This cascade of molecular events boosts glycolytic flux by elevating glucose consumption, lactate production, and ATP synthesis. The resulting surge in glycolytic activity, prompted by BHLHE40 and GRIN2D, not only sustains tumor cell survival and proliferation but also correlates with poor histological differentiation and more aggressive tumor phenotypes, thus contributing to the overall malignancy of GC.

Another study demonstrated that *H. pylori* infection modulates glycolysis in GC cells via the upregulation of MyoD family inhibitor (MDFI) and subsequent activation of the Wnt/β-catenin signaling pathway [47]. The increased MDFI expression enhances the glycolytic machinery, promoting key processes such as glucose uptake, lactate production, and ATP generation. These alterations in cellular metabolism reinforce the tumor’s survival and growth advantages. Moreover, *H. pylori*-infected gastric epithelial cells exhibit elevated expression of glycolysis-pivotal enzymes such as Lon protease 1 (Lonp1) and pyruvate kinase M2 (PKM2). Lonp1 is essential for maintaining mitochondrial quality via activation of the mitochondrial unfolded protein response (UPRmt). In the context of *H. pylori* infection, Lonp1 is upregulated through the action of hypoxia-inducible factor 1α (HIF-1α), further accelerating glycolysis and cell proliferation [48, 49]. Similarly, PKM2, a critical enzyme in the final step of glycolysis, is upregulated in response to the H. pylori-associated CagA. The activation of the Erk signaling pathway by CagA enhances PKM2 expression, thereby driving glycolytic activity and promoting serine-dependent cancer cell proliferation. Silencing PKM2 results in a significant inhibition of glycolysis, substantially reducing tumor cell proliferation, migration, and metastasis [50].

Beyond its influence on enzymes, CagA also disrupts the normal regulation of metabolism by inhibiting sirtuin 3 (SIRT3) and stabilizing HIF-1α, thereby amplifying glycolysis [51]. In addition, the Akt pathway is activated, further promoting glycolysis. Inhibition of glycolysis or the Akt pathway has been shown to sensitize GC cells to 5-fluorouracil (5-Fu), highlighting potential therapeutic targets for overcoming resistance in both in vitro and in vivo models [52].

**Fig. 1:**
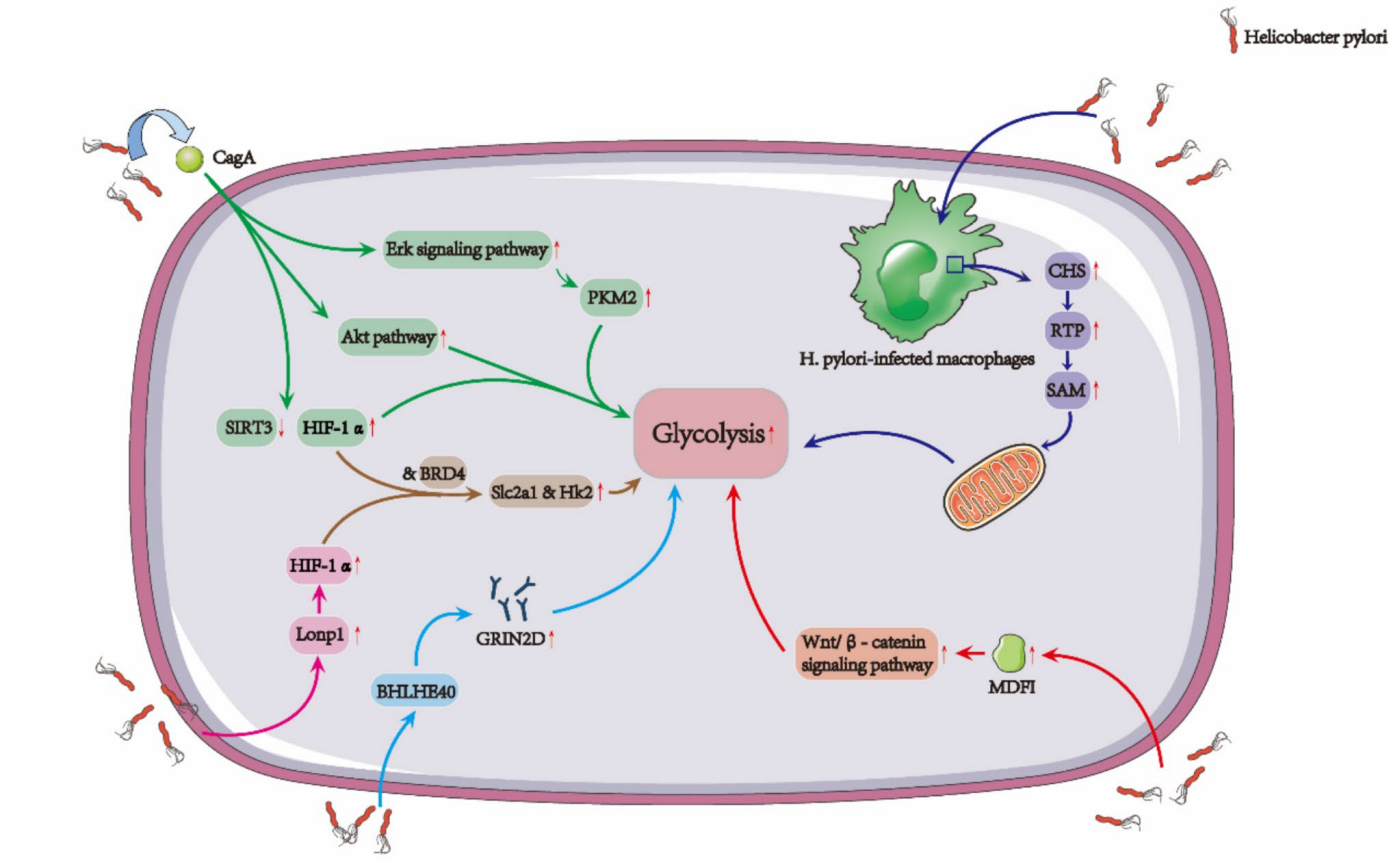
*H. pylori* promotes glycolysis via multiple pathways, enhancing tumor proliferation, inflammation, and immune evasion in gastric cancer

In addition to its direct impact on GC cells, *H. pylori* also influences the immune microenvironment, amplifying inflammatory responses and contributing to tumor progression. For instance, cystathionine γ-lyase (CTH), activated in *H. pylori*-infected macrophages, drives the mammalian reverse transsulfuration pathway (RTP), which promotes S-Adenosylmethionine (SAM) metabolism and enhances mitochondrial function. This activation, in turn, drives glycolysis, exacerbating inflammation and promoting a vicious cycle that favors tumorigenesis [53]. Furthermore, *H. pylori*-induced immune responses and the hypoxic conditions within the tumor niche activate HIF-1α, which collaborates with BRD4 to upregulate glycolysis-related genes such as Slc2a1 (Solute Carrier Family 2 Member 1) and Hk2 (hexokinase 2) [54] (Fig. 1). Together, these molecular interactions enhance glucose uptake and metabolic enzyme activity, ensuring a constant supply of energy to sustain the aggressive metabolic needs of GC cells.

Collectively, the metabolic alterations induced by *H. pylori* infection, particularly the persistent upregulation of glycolysis, not only provide rapid energy and biosynthetic intermediates to sustain abnormal cellular proliferation but also profoundly reshape the cellular and tissue microenvironment, thereby actively driving the initiation and progression of GC. Specifically, enhanced glycolytic flux leads to excessive lactate accumulation and subsequent acidification of the tumor microenvironment. This acidic milieu directly suppresses the function of immune effector cells such as T cells and NK cells, thereby promoting immune evasion. Moreover, it reprograms the metabolism of infiltrating immune cells toward immunosuppressive phenotypes, further weakening antitumor immunity. In parallel, extracellular acidification facilitates matrix degradation and remodeling, enhances the invasive and metastatic potential of tumor cells, and promotes angiogenesis by activating pro-angiogenic signaling pathways, collectively providing sustained support for malignant transformation [55, 56].

Importantly, the activation of oncogenic signaling pathways such as PI3K/AKT/mTOR and RAS/RAF/MEK/ERK not only sustains glycolytic activation but also regulates transcription factors including HIF-1α and c-Myc to upregulate oncogenes and pro-tumor cytokines, thereby intensifying metabolic reprogramming [57, 58]. This bidirectional reinforcement between altered glucose metabolism and oncogenic signaling establishes a positive feedback loop that tightly links glycolytic reprogramming with gastric carcinogenesis. In other words, aberrant glycolysis is not merely a metabolic adaptation but also a key driving force for malignant transformation, integrating energy supply, cell fate regulation, and tumor microenvironment remodeling to directly promote the initiation and progression of GC.

### *H. pylori* and lipid metabolism

Lipid metabolism stands as a cornerstone in the complex web of tumorigenesis and cancer progression. When dysregulated, lipid metabolism does more than merely fuel tumor growth; it crafts a metabolic landscape that nurtures a favorable microenvironment for cancer to take root, thrive, and spread. The link between lipid metabolism and cancer is multifaceted, with alterations in lipid pathways not only sustaining tumor cell proliferation but also paving the way for metastasis and the formation of secondary tumors in distant organs [59, 60].

Cancer cells acquire fatty acids (FAs) through lipid catabolism, promoting de novo FA synthesis and enhancing lipid uptake. These FAs fuel critical processes in tumorigenesis—energy production, membrane synthesis, and signaling pathways that orchestrate cellular growth. A pivotal enzyme in this process is ATP-citrate lyase (ACLY), which catalyzes the conversion of citrate to acetyl-CoA, a key building block for FA synthesis. This reaction provides a major energy source for the growth and metabolism of cancer cells, serving as the foundation for FA synthesis [61]. Intriguingly, studies have shown that *H. pylori* infection increases ACLY gene expression, further amplifying acetyl-CoA production. This upregulation deepens the interaction between *H. pylori* and gastric epithelial cells, setting the stage for gastritis, epithelial damage, and over time, the development of gastroduodenal ulcers and gastric carcinoma [62].

Beyond FA metabolism, *H. pylori* infection also exerts a profound influence on sphingolipid metabolism, another pivotal pathway in GC progression. Specifically, the bacterium induces glucosylceramidase beta 1 (GBA1) expression through promoter demethylation, thereby enhancing sphingolipid metabolism. The elevated GBA1 levels in infected cells help mitigate lipid peroxidation, protect against ferroptosis, and stabilize antioxidant defenses, including glutathione and the reduction of malondialdehyde. This shift in the metabolic landscape serves the dual purpose of promoting GC cell proliferation and facilitating tumor growth by counteracting the oxidative stress that often accompanies cancer progression [26].

Moreover, recent research highlights that *H. pylori* infection elevates the expression of phosphorylase kinase G2 (PHKG2), a key regulator of lipid metabolism. PHKG2 enhances the activity of arachidonate 5-lipoxygenase (ALOX5), a crucial enzyme involved in lipid peroxidation. The *H. pylori*-induced increase in PHKG2 and ALOX5 paradoxically promotes GC progression by altering the TME and lipid metabolism [63]. This elevated oxidative stress triggers genetic instability, chronic inflammation, and promotes immune evasion—factors that collectively accelerate GC growth and metastasis.

Cholesterol metabolism is another vital pillar in the metabolic reprogramming initiated by *H. pylori*. Studies by Zhang et al. demonstrated that the infection induces the translocation of Cytochrome P450 Family 11 Subfamily A Member 1 (CYP11A1) from the mitochondria to the cytoplasm, a process mediated by the CagA protein. This shift results in reduced mitochondrial levels of CYP11A1 and an accumulation of cholesterol within the mitochondria, promoting mitophagy, which promotes GC progression by increasing cellular resistance [64]. Adding another layer of complexity, Kuo’s study revealed that during *H. pylori* infection, the bacterium binds directly to cholesterol present in host cell membranes, using it as a critical resource for its own colonization and pathogenesis. This interaction leads to the depletion of cholesterol from lipid rafts, which are essential for proper cell signaling. This depletion creates a cascade effect, recruiting key inflammatory mediators such as IL-33 and its receptor ST-2 into the lipid raft regions. The aggregation of these mediators intensifies downstream signaling pathways, resulting in the production of pro-inflammatory cytokines, such as IL-8, which in turn promotes leukocyte infiltration into gastric tissues, worsening the inflammatory environment during infection [65].

**Fig. 2:**
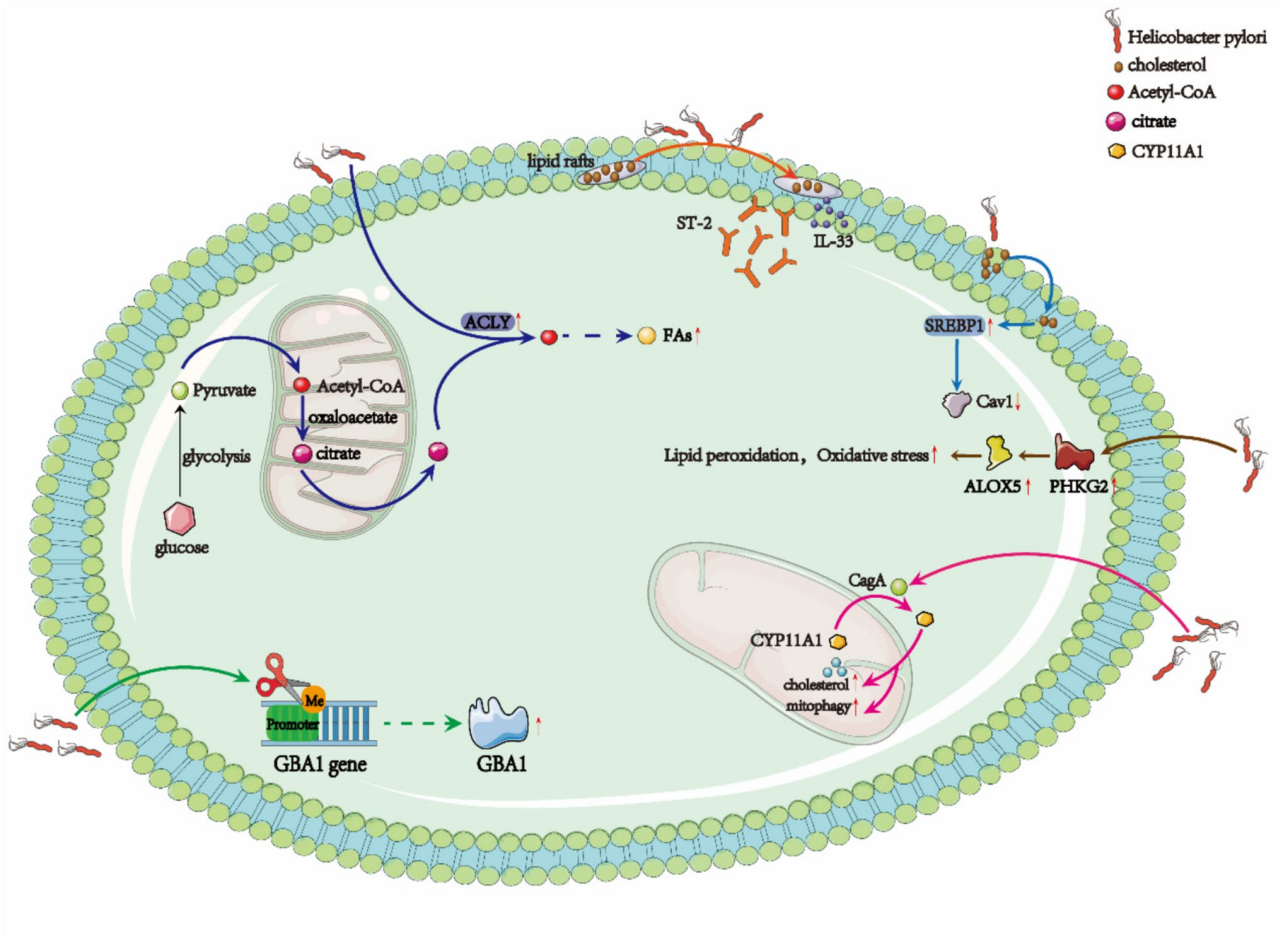
*H. pylori*-induced lipid metabolic reprogramming facilitates tumor growth, ferroptosis resistance, and immune dysregulation in gastric cancer

The cholesterol depletion also activates the sterol regulatory element-binding protein 1 (SREBP1), a transcription factor central to lipid metabolism. Once activated, SREBP1 drives the downregulation of caveolin-1 (Cav1), a protein integral to immune cell function and cellular interactions. The loss of Cav1 impairs immune responses by disrupting macrophage function and enhancing immune cell infiltration into gastric tissue. This immune dysfunction amplifies the inflammatory response and contributes to the persistence of GC [66]. Notably, SREBP1 is overexpressed in some digestive system cancers, including pancreatic and colon cancers, where it has been shown to promote tumor proliferation and invasion [67, 68] (Fig. 2).

Therefore, *H. pylori*-induced lipid metabolic reprogramming not only facilitates gastric carcinogenesis by supplying the energy and biosynthetic precursors required for tumor cell proliferation, but also regulates key cellular processes that support malignant transformation. Enhanced fatty acid synthesis and altered sphingolipid metabolism provide essential components for membrane biosynthesis, signaling molecules, and energy storage, thereby sustaining rapid cell division and continuous proliferation. At the same time, these alterations markedly enhance cellular resistance to oxidative stress and ferroptosis, enabling tumor cells to survive and expand under metabolic pressure and inflammatory microenvironmental stressors [67]. Furthermore, disruption of cholesterol homeostasis and lipid raft integrity directly interferes with membrane-associated signal transduction, leading to hyperactivation of pro-inflammatory signaling cascades and impaired communication among immune cells, ultimately fostering an immunosuppressive microenvironment. More importantly, these lipid metabolic alterations not only affect membrane fluidity and signaling efficiency but also modulate critical pathways such as PI3K/AKT and MAPK, thereby amplifying oncogene activation and inflammatory responses. Through this metabolic reprogramming, tumor cells acquire multiple advantages in terms of energy supply, structural support, and signaling capacity, which in turn enhance their angiogenic, migratory, and invasive potential.

In summary, lipid metabolic reprogramming not only shapes an immunosuppressive and pro-inflammatory microenvironment conducive to tumor development, but also reinforces oncogenic signaling networks, thereby establishing a direct mechanistic link between metabolic reprogramming and malignant transformation. Ultimately, lipid metabolism alterations drive gastric cancer initiation and progression through the dual mechanisms of intrinsic cellular adaptation and microenvironmental modulation.

### *H. pylori* and amino acid metabolism

Amino acids are not merely the foundational building blocks of life, but also serve as dynamic regulators in cellular metabolism, influencing a variety of physiological processes beyond their well-known role in protein synthesis [69]. These metabolic networks exert complex, pleiotropic effects on cellular homeostasis, orchestrating a balance between growth, survival, and adaptation. Increasingly, evidence reveals that *H. pylori* exploits host amino acid metabolism to its advantage, subtly manipulating key metabolic pathways through its virulence factors. In particular, the bacterium targets glutamine, tryptophan, and arginine pathways, creating a tumor-friendly microenvironment that not only fuels bacterial persistence but also evades the body’s immune defenses, thereby facilitating gastric carcinogenesis.

A central player in this metabolic reprogramming is the CagA, which is translocated into host cells via the T4SS. Once inside the cell, CagA sparks carcinogenic signaling by activating the PI3K/AKT pathway, a crucial driver of cellular survival and proliferation [70, 71]. This signaling cascade, in turn, upregulates the expression of amino acid transporters such as L-type amino acid transporter 1 (LAT1) and alanine–serine–cysteine transporter 2 (ASCT2), enhancing the uptake of glutamine and other essential amino acids. This influx of amino acids supplies the building blocks necessary for the unchecked growth and division of epithelial cells [72, 73]. In contrast, the VacA toxin demonstrates a dual role in modulating host metabolism. On one hand, VacA’s pore-forming activity induces mitochondrial depolarization and inhibits autophagic flux, leading to the accumulation of amino acids in the cytosol that sustain neoplastic cell survival [74]. On the other hand, VacA also suppresses mammalian target of rapamycin complex 1 (mTORC1), creating a local amino acid deprivation that triggers compensatory autophagy, all while simultaneously disrupting mitochondrial bioenergetics [73]. This complex interplay between nutrient availability and metabolic stress undermines host antimicrobial defenses, setting the stage for persistent *H. pylori* infection and, ultimately, GC.

Among the most intriguing metabolic shifts induced by *H. pylori* is the catabolism of tryptophan. The bacterium activates the kynurenine pathway, a process mediated by cGAS-IRF3-dependent upregulation of kynurenine aminotransferase II (KAT2), leading to the conversion of tryptophan into immunosuppressive kynurenine derivatives. This metabolic diversion not only promotes immune evasion by fostering the differentiation of Treg cells and the exhaustion of cytotoxic T cells, but also contributes to chemoresistance [75–77]. A particularly notable metabolite in this pathway is xanthurenic acid (XA), which accumulates in the system and transcriptionally activates Caudal Type Homeobox 2 (CDX2), a master regulator of intestinal metaplasia in gastric epithelial cells. This epigenetic remodeling, driven by kynurenine metabolism, adds another layer of complexity to the tumorigenic transformation [9].

Meanwhile, glutamine metabolism takes center stage in the *H. pylori*-induced reprogramming of the gastric environment. The bacterium’s gamma-glutamyl transferase (GGT) depletes glutamine, disrupting the production of α-ketoglutarate (α-KG), a critical metabolite required for proper TCA cycle flux. As a result, this depletion not only impairs energy production but also triggers the accumulation of ammonia and reactive oxygen species (ROS), which in turn induces global histone hypermethylation (H3K9me3/H3K27me3) [78]. This epigenetic alteration activates pro-proliferative gene programs, enhancing the growth potential of gastric epithelial cells. Concurrently, glutamine-starved mesenchymal stem cells exhibit increased self-renewal capacity and heightened expression of pluripotency markers, contributing to tumor stroma remodeling and further fueling the carcinogenic process [79].

**Fig. 3:**
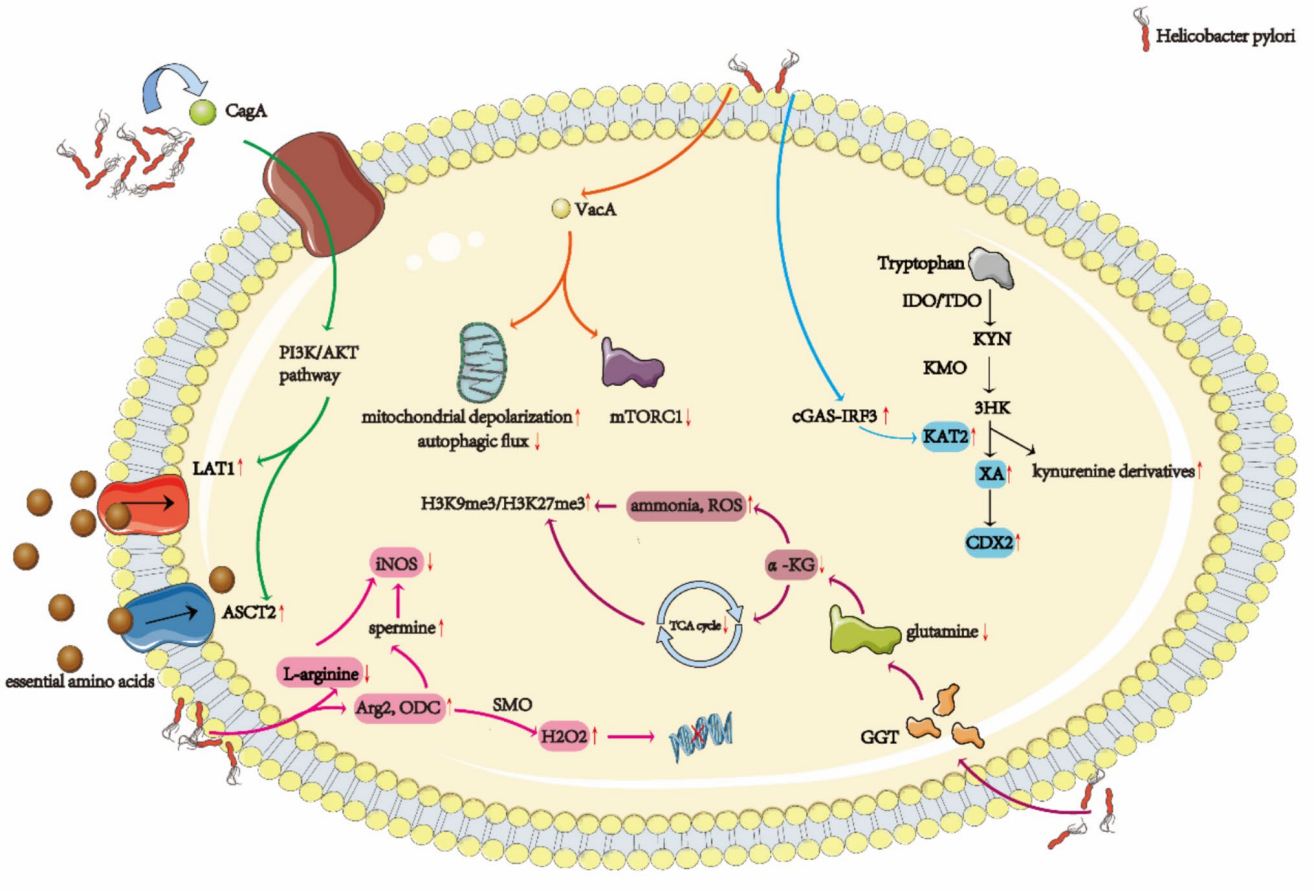
*H. pylori*-induced reprogramming of amino acid metabolism facilitates immunosuppression and epigenetic remodeling to promote gastric cancer progression

Equally strategic is the bacterium’s manipulation of arginine metabolism, which exemplifies *H. pylori*’s immune evasion tactics. Through the action of bacterial arginase, *H. pylori* competitively depletes l-arginine, a critical substrate for the inducible nitric oxide synthase (iNOS) pathway that is normally responsible for producing nitric oxide (NO), a key mediator of immune responses. In parallel, *H. pylori* upregulates host arginase II (Arg2) and ornithine decarboxylase (ODC), rerouting the residual arginine toward the synthesis of polyamines. Elevated levels of spermine, a polyamine derivative, suppress iNOS activity and generate genotoxic hydrogen peroxide (H₂O₂) through the action of spermine oxidase (SMO). This dual mechanism—combining immune suppression with oxidative DNA damage—creates a perfect storm, tipping the scales toward malignant transformation and tumor progression [80] (Fig. 3).

In summary, *Helicobacter pylori* strategically reprograms key amino acid metabolic pathways, including those of tryptophan, glutamine, and arginine, not only to fulfill its own bioenergetic and nutritional demands but also to profoundly reshape the gastric microenvironment. This metabolic reprogramming exerts multilayered effects on host cells. First, dysregulated tryptophan metabolism activates the indoleamine-2, 3-dioxygenase (IDO) pathway, thereby promoting immune tolerance, suppressing T-cell proliferation and effector functions, and ultimately impairing antitumor immune surveillance [81]. In addition, tryptophan-derived metabolites can act as signaling molecules that regulate the aryl hydrocarbon receptor (AhR) pathway, inducing epigenetic and transcriptional reprogramming of epithelial cells and increasing their susceptibility to malignant transformation. Second, glutamine metabolic reprogramming provides tumor cells with nitrogen and tricarboxylic acid cycle intermediates, thereby sustaining rapid proliferation as well as nucleotide and lipid biosynthesis. As an essential cellular “fuel,” glutamine also supports glutathione synthesis, enhancing resistance to oxidative stress and enabling cell survival under metabolic pressure and inflammatory microenvironments [82]. Moreover, abnormal arginine metabolism, primarily through the regulation of nitric oxide (NO) production, plays a pivotal role in cell signaling, angiogenesis, and immune modulation. *H. pylori*-induced arginine dysregulation promotes the activation of immunosuppressive myeloid cells, thereby impairing T-cell function and fostering an immune-evasive microenvironment. Simultaneously, excess or imbalanced NO contributes to DNA damage and the accumulation of mutations, directly driving genomic instability and malignant progression [83].

Collectively, amino acid metabolic reprogramming not only undermines immune surveillance but also facilitates epigenetic alterations, sustains proliferative and stress-adaptive signaling pathways, and promotes genomic instability and immune evasion. These processes synergistically drive the transition of gastric mucosa from chronic inflammation to epithelial metaplasia and, ultimately, to malignant transformation [80]. Thus, dysregulated amino acid metabolism constitutes a central mechanistic link between *H. pylori* infection and gastric carcinogenesis.

## Targeting metabolic reprogramming and GC therapy

Targeting metabolic reprogramming has swiftly emerged as a cornerstone strategy in the treatment of GC, as malignant cells exploit altered metabolic pathways to fuel relentless proliferation and evade programmed cell death. In this context, the disruption of key enzymes and signaling cascades within these reprogrammed networks is seen as a promising therapeutic approach, offering potential for precision medicine with enhanced efficacy [84–86]. The following sections delineate therapeutic interventions targeting glycolysis, amino acid metabolism, and lipid metabolism, supported by preclinical and clinical insights.

### Targeting glycolysis

At the very heart of aerobic glycolysis lies HK2, an enzyme that catalyzes the conversion of glucose into glucose-6-phosphate (G-6-P), establishing its dominance over other isoforms in promoting GC glycolysis [87]. The pharmacological modulation of HK2 has emerged as a promising avenue for therapeutic intervention. Peroxisome proliferator-activated receptor alpha (PPARα), a key regulator in GC, is often overexpressed and strongly correlated with poor prognosis. Fenofibrate restores mitochondrial function via the PPARα pathway, promotes Carnitine Palmitoyltransferase 1 (CPT1)-mediated fatty acid oxidation and activation of the AMP-activated protein kinase (AMPK) pathway, and suppresses HK2, ultimately suppressing cell proliferation and inducing apoptosis [88]. Similarly, baicalein inhibits glycolytic enzymes, including hexokinase 2 (HK2), lactate dehydrogenase A (LDH-A), and pyruvate dehydrogenase kinase 1 (PDK1), by activating phosphatase and tensin homolog (PTEN) and suppressing Akt/HIF-1α signaling, thereby reducing glycolytic flux and sensitizing GC cells to overcome 5-FU resistance in GC cells [89]. Licochalcone A (LicA) also demonstrates promise by downregulating HK2 expression through blockade of the Akt pathway, thereby impairing glucose utilization and delaying tumor progression [90]. In trastuzumab-resistant GC, metformin suppresses HK2-dependent glycolysis by inhibiting period circadian regulator 1 (PER1)-mediated circadian regulation of HK2, thereby restoring trastuzumab sensitivity and inhibiting tumor growth [91]. More and more evidence further supports the use of natural compounds such as paeonol [92], the active ingredient DT-13 of ophiopogon [93], and targeted inhibitors to enhance chemotherapy sensitivity and reduce tumor invasion of GC by disrupting HK2 and related pathways.

PKM2 is an enzyme crucial for the final step in glycolysis. Unlike other isoforms, PKM2 is primarily found in the cytoplasm, where it not only catalyzes the conversion of phosphoenolpyruvate to pyruvate but also presents another actionable target for GC therapy [94]. The phosphatidylinositol 3-kinase (PI3K) inhibitor LY294002 effectively suppresses PKM2 expression, thereby attenuating glycolytic activity, reducing lactate dehydrogenase activity and lactate production, and ultimately inhibiting GC cell proliferation while promoting apoptosis [95]. In addition to PKM2, other glycolytic enzymes also play essential roles in gastric cancer progression. LDH, which comprises subunits such as LDH-A, LDH-B, and LDH-C, is integral to both anaerobic glycolysis and gluconeogenesis, with elevated LDH expression often linked to poor prognosis in various cancers [96]. In GC, topoisomerase 1 mitochondrial (TOP1MT) deficiency has been shown to enhance aerobic glycolysis by upregulating LDH-A, promoting glucose consumption and lactate production, and further driving epithelial–mesenchymal transition (EMT). Targeting LDH-A could reduce TOP1MT gene expression and inhibit GC development [97].

Beyond direct enzyme inhibition, alternative approaches have gained traction. 2-Deoxyglucose (2-DG), a hexokinase antagonist, when combined with metformin, was observed to attenuate *H. pylori*-induced premalignant lesions like intestinal metaplasia in a Mongolian gerbil model, potentially through suppression of glycolysis [98]. Alternatively, YC-1, a HIF-1α inhibitor, reprograms glucose metabolism in hypoxic GC cells by suppressing HIF-1α-mediated glycolytic gene expression, thereby shifting energy production from glycolysis to oxidative phosphorylation (OXPHOS). This metabolic shift leads to excessive reactive oxygen species (ROS) accumulation, triggering apoptosis and markedly inhibiting tumor growth in xenograft models [99]. M2 macrophage-derived exosomal metastasis-associated lung adenocarcinoma transcript 1 (MALAT1) promotes GC progression by activating β-catenin and HIF-1α signaling to enhance glycolysis, while MALAT1 silencing suppresses tumor growth and improves chemosensitivity [100]. Furthermore, integrating glycolysis inhibitors with immune checkpoint blockade has shown promise in enhancing antitumor immunity by modulating the tumor microenvironment in advanced GC [101, 102].

### Targeting lipid metabolism

Lipid metabolic reprogramming is yet another central feature in GC progression. Bioinformatics analyses reveal that lipid metabolism not only shapes the tumor immune microenvironment (TIME) but also impacts patient prognosis. Specifically, low lipid metabolism expression correlates with reduced immune cell infiltration, while key metabolic pathways such as arachidonic acid metabolism govern immune responses and cell migration [103]. Among the key enzymes driving lipid metabolic reprogramming, ATP-citrate lyase (ACLY) plays a central role, which fuels lipid metabolism by increasing intracellular FAs and triglyceride levels. This, in turn, activates the AKT/mTOR signaling pathway, promoting cancer cell proliferation. Targeting ACLY is therefore seen as a promising approach for GC treatment [104]. In addition, the lipolysis-stimulated lipoprotein receptor (LSR) promotes the uptake of lipoproteins into GC cells. This leads to lipid droplet accumulation and increased fatty acid β-oxidation, driving tumor growth in GC. Clinically, high LSR expression and lipid droplet formation are linked to poor prognosis; blocking β-oxidation with Etomoxir can reverse these effects, reducing cell proliferation in vitro and tumor growth in vivo [105]. The latest research shows that natriuretic peptide receptor 1 (NPR1) promotes gastric cancer metastasis by activating lipolysis and enhancing fatty acid-driven oxidative phosphorylation (OXPHOS), while NPR1 silencing suppresses metastasis, highlighting it as a potential lipid metabolism-targeted therapy [106].

Furthermore, the sterol O-acyltransferase 1 (SOAT1) inhibitor Avasimibe disrupts lipid metabolism by reducing cholesterol ester synthesis, impairing lymphangiogenesis, and inhibiting tumor proliferation through downregulation of SREBP1/2 and VEGF-C [107]. HDAC inhibitors (HDACi) remodel lipid metabolism by upregulating key enzymes such as acyl-CoA synthetase long-chain family member 4 (ACSL4) and P450 oxidoreductase (POR), while downregulating solute carrier family 7 member 11 (SLC7A11), which triggers lipid peroxidation accumulation and ultimately induces ferroptosis, thus inhibiting GC progression [108]. In addition, the metabolically hostile TIME fosters immunosuppression through nutrient competition. For instance, Yang et al. found that inhibiting cholesterol esterification with Avasimibe enhances CD8 + T-cell cytotoxicity by increasing membrane cholesterol, boosting T-cell receptor signaling, and promoting immune synapse formation. This metabolic reprogramming enhances antitumor immunity and, when combined with anti-PD-1 therapy, shows improved therapeutic efficacy [109].

Thus, combining metabolic inhibitors with immune checkpoint inhibitors (ICIs) such as anti-PD-1/PD-L1 antibodies holds substantial promise for enhancing antitumor immunity and advancing GC treatment [110].

### Targeting amino acid metabolism

Moving into the realm of amino acid metabolism, the metabolism of glutamine plays a pivotal role in the growth of GC, with transporters like alanine–serine–cysteine transporter 2 (ASCT2) and enzymes such as glutamine synthetase (GS) determining the tumor’s dependence on both exogenous and endogenous glutamine. Targeting both ASCT2 and GS has been shown to enhance therapeutic efficacy in GC treatment [111]. In addition, glutaminase inhibitors like CB-839 disrupt glutaminolysis, thus inhibiting tumor growth and suppressing GC progression [112, 113].

Beyond glutamine, tryptophan metabolism-associated genes, such as ECHS1 and ALDH2, play a key role in shaping the tumor microenvironment and modulating immune responses, and have emerged as potential targets for personalized immunotherapy [114]. A recent study has shown that Indoleamine 2,3-dioxygenase 1 (IDO1) promotes the conversion of tryptophan into the immunosuppressive metabolite kynurenine. Targeting IDO1 can block this process, restore T-cell function, and enhance the efficacy of Claudin 18.2 (CLDN18.2)-chimeric antigen receptor T-cell (CAR-T) therapy in GC [115].

Similarly, alterations in L-arginine metabolism, particularly changes in the expression of key enzymes such as argininosuccinate lyase (ASL), protein arginine methyltransferases (PRMTs), and dimethylarginine dimethylaminohydrolases (DDAHs), contribute to GC progression and offer promising avenues for diagnostic and therapeutic intervention [116]. In addition, the dual arginase inhibitor OATD-02 restores intratumoral L-arginine levels by blocking arginase 1 (ARG1) and arginase 2 (ARG2)-mediated depletion, thereby reducing polyamine biosynthesisand enhancing antitumor immune responses with increased T-cell infiltration in tumors; this strategy may be used to enhance the efficacy of immunotherapy for gastric cancer and improve the antitumor outcomes [117].

Overall, multi-omics analyses have helped refine treatment strategies by revealing GC subtypes with distinct metabolic traits and clinical outcomes. Some tumors, such as those dominated by glycolysis, tend to have a poor prognosis, yet they also show greater sensitivity to chemotherapy, whereas glutamate-dependent subtypes are more likely to benefit from immunotherapy [118]. These findings emphasize that treatment should be tailored to the metabolic profile of each tumor, a strategy that could greatly improve patient clinical outcomes.

## Conclusion

*H. pylori* infection has long been recognized as a key factor in the development and progression of gastric cancer. Not content with merely setting the stage, *H. pylori* actively reshapes the metabolic mechanisms of gastric tumor cells, thereby promoting the malignant progression of gastric cancer.

*H. pylori* disrupts a constellation of critical metabolic pathways, from the rapid-fire energy production of glycolysis to the complex choreography of lipid metabolism and the nuanced regulation of amino acid synthesis. These alterations are profound: they not only furnish tumor cells with the means to survive and proliferate but also arm them with strategies to evade immune detection and accelerate metastasis. In essence, the metabolic reprogramming spurred by *H. pylori* equips tumor cells with an extraordinary adaptability, enabling them to thrive even under the most adverse conditions. Understanding how *H. pylori* infection drives this metabolic reprogramming is crucial to uncovering the molecular mechanisms that underpin GC development.

Future research should focus on specific metabolic pathways, especially glycolysis, lipid metabolism, and amino acid metabolism. By addressing the metabolic disorders caused by *H. pylori*, theoretical support is provided for the precise and personalized treatment of GC. In addition, combining targeted metabolic reprogramming with *H. pylori* eradication therapy may further improve treatment outcomes in GC, representing a promising avenue for future research and clinical translation.

## Abbreviations

*H. pylori*: *Helicobacter pylori*
GC: Gastric cancer
WHO: World health organization
MALT: Mucosa-associated lymphoid tissue
CagA: Cytotoxin-associated gene A
VacA: Vacuolating cytotoxin A
T4SS: Type IV secretion system
CagPAI: Cag pathogenicity island
TRPML1: Transient receptor potential mucolipin 1
TME: Tumor microenvironment
ATP: Adenosine triphosphate
GRIN2D: Glutamate ionotropic receptor N-methyl-D-aspartate type subunit 2D
BHLHE40: Class E basic helix–loop–helix protein 40
MDFI: MyoD family inhibitor
Lonp1: Lon protease 1
PKM2: Pyruvate kinase M2
UPRmt: Mitochondrial unfolded protein response
SIRT3: Metabolism by inhibiting sirtuin 3
5-Fu: 5-Fluorouracil
CTH: Cystathionine γ-lyase
RTP: Reverse transsulfuration pathway
SAM: S-Adenosylmethionine
Slc2a1: Solute carrier family 2 member 1
HK2: Hexokinase 2
FA: Fatty acid
ACLY: ATP-citrate lyase
GBA1: Glucosylceramidase beta 1
PHKG2: Phosphorylase kinase G2
ALOX5: Arachidonate 5-lipoxygenase
CYP11A1: Cytochrome P450 family 11 subfamily a member 1
SREBP1: Sterol regulatory element-binding protein 1
Cav1: Caveolin-1
LAT1: L-type amino acid transporter 1
ASCT2: Alanine–serine–cysteine transporter 2
mTORC1: Mammalian target of rapamycin complex 1
KAT2: Kynurenine aminotransferase II
XA: Xanthurenic acid
CDX2: Caudal type homeobox 2
GGT: Gamma-glutamyl transferase
α-KG: α-Ketoglutarate
NOS: Inducible nitric oxide synthase
NO: Nitric oxide
Arg2: : Arginase II
ODC: Ornithine decarboxylase
H₂O₂: Hydrogen peroxide
SMO: Spermine oxidase
G-6-P: Glucose-6-phosphate
PPARα: Peroxisome proliferator-activated receptor alpha
CPT1: Carnitine palmitoyltransferase 1
AMPK: AMP-activated protein kinase
LDH: Lactate dehydrogenase
LicA: Licochalcone A
TOP1MT: Topoisomerase 1 mitochondrial
EMT: Epithelial–mesenchymal transition
2-DG: 2-Deoxyglucose
OXPHOS: Oxidative phosphorylation
ROS: Reactive oxygen species
ASCT2: Alanine–serine–cysteine transporter 2
GS: Glutamine synthetase
TIME: Tumor immune microenvironment
LSR: Lipoprotein receptor
SOAT1: Sterol o-acyltransferase 1
HDACi: HDAC inhibitors
ACSL4: Acyl-CoA synthetase long-chain family member 4
POR: P450 oxidoreductase
SLC7A11: Solute carrier family 7 member 11
ICIs: Immune checkpoint inhibitors
ASL: Argininosuccinate lyase
PRMTs: Protein arginine methyltransferases
DDAHs: Dimethylarginine dimethylaminohydrolases
PER1: Period circadian regulator 1
MALAT1: Metastasis-associated lung adenocarcinoma transcript 1
